# Help seeking in school by Israeli Arab minority adolescents with emotional and behavioral problems: results from the Galilee Study

**DOI:** 10.1186/s13584-016-0109-0

**Published:** 2016-12-01

**Authors:** Raida Daeem, Ivonne Mansbach-Kleinfeld, Ilana Farbstein, Raseem Khamaisi, Anneke Ifrah, Ahmad Sheikh Muhammad, Sylvana Fennig, Alan Apter

**Affiliations:** 1 Sackler Faculty of Medicine, Tel Aviv University, Tel Aviv, Israel; 2 Child and Adolescent Mental Health Department, Ziv Medical Center, Zefat, 13100 Israel; 3 The Feinberg Child Study Center, Schneider Medical Center for Children in Israel, Petach Tikvah, 49202 Israel; 4 The Faculty of Medicine in the Galilee, Bar Ilan University, Safed, Israel; 5 Department of Geography and Environmental Studies, University of Haifa, Haifa, 31905 Israel; 6 Israel Center for Disease Control, Ministry of Health, Ramat Gan, Israel; 7 The Galilee Society, The Arab National Society for Health Research and Services, PO Box 330, Shefa Amr, Israel 20200; 8 Department of Child and Adolescent Psychiatry, The Feinberg Child Study Center, Schneider Children’s Medical Center of Israel, 14 Kaplan St, Petach Tikva, 49202 Israel

**Keywords:** Adolescents, Mental health, School help-seeking, Israeli Arab minority, Mental Health Reform Israel, SDQ, Muslim, Druze

## Abstract

**Background:**

Many distressed minority adolescents with little access to professional mental health services use teachers and school counselors as their main consultation sources. This paper presents data from the Galilee study on factors that may increase the probability of adolescents’ help-seeking in school and discusses the needed linkage between the school mental health services and those provided by other agencies, in the framework of the Mental Health Reform in Israel.

**Methods:**

This cross-sectional survey included 1639, 9th grade students living in 5 Arab localities in the Galilee in northern Israel, representative of the Muslim and Druze populations. The study was carried out in two stages: in the screening stage, the Strengths and Difficulties Questionnaire (SDQ) was completed in the classroom. During the follow-up stage 704 students were selected and interviewed at home regarding service use in school and wellbeing at home. Their mothers were interviewed as well providing information on sociodemographic traits of the family. Total response rate was 69.3 % during the screening stage and 84.4 % during the follow-up. Students were categorized according to their SDQ scores and all students in the higher 25th percentile (high risk) and a simple systematic sample without replacement of those in the lower 75 % (low risk) were included in the follow-up study.

**Results:**

Significantly more high risk than low risk students reported having felt the need to seek professional help (14.0 and 6.5 % respectively) and more high risk than low risk consulted a school source (27.1 and 15.2 %, respectively). Bivariate analyses show that among Muslim adolescents more high risk than low risk consulted a school source (30 vs. 16.2 % respectively) and among high risk students more Muslim than Druze sought help from a school source (30 vs. 18 %). Higher consultation rates were found among adolescents who felt uncomfortable at home, than among those who felt very comfortable. Binary logistic regression showed that for high risk adolescents, only religion remained significantly associated with help-seeking in school: Muslim students were 2 times more likely to seek help than Druze students. In the low-risk group, students who do not feel comfortable at home were 3.3 times more likely to seek help than those who feel comfortable at home. The main sources of consultation for both risk groups were the school counselor and the grade teacher.

**Conclusions:**

A constellation of factors may be associated with help-seeking in school by minority Israeli Arab adolescents: they are students at higher risk for an emotional or behavioral disorder, they have more socio-economic hardship, they feel less comfortable at home and they are more likely to live in the larger Muslim cities. Adolescents with less family support and particularly those not classified as being at high risk, are more likely to seek help from school counselors and teachers. The school staff may need additional training to care for the mental health needs of students. There is a need to integrate the school mental health services with the other government agencies that provide services to children and adolescents.

## Background

It is known that a relatively small percentage of distressed adolescents seek professional mental health care in the community, as most usually prefer to consult family members or friends [1–3]. It is also known that minorities in general have lower rates of mental health services utilization than the majority population, due to poor availability and accessibility of services in the community [4–7]. However, among minority adolescents, rates of help-seeking in school are higher than among majority adolescents [1, 8] due to the ability of the school-based services to be available at all times, the low cost and the fact that there is no need for special transportation [9]. There is also a language and cultural fit between students and school staff, and often a rapport and trust in teachers and school counselors [5, 6, 9, 10]. In addition, school consultation may be perceived as a normative act, whereas turning to professional mental health sources, particularly psychologists, may be perceived as stigmatic [11]. Some studies show that adolescents perceive school staff as more acquainted with their lives and as better able to understand their difficulties than mental health professional sources and that students believe school staff will continue to value them despite the disclosure of their weaknesses [12, 13].

The Israel Survey of Mental Health among Adolescents (ISMEHA), carried out in 2004–2005, found that among Israeli Arabs, 51 % of adolescents with a mental disorder consulted a school source, as compared to only 30 % of Jewish majority adolescents [8]. One of the instruments used in the ISMEHA for the assessment of emotional and behavioral problems was the Strengths and Difficulties Questionnaire (SDQ), a screening instrument designed for evaluating social, emotional and behavioral functioning in 4–17 year old children and adolescents [14, 15]. Studies have shown that SDQ scores reflect genuine differences in child mental health [16], provide accurate estimates of disorder prevalence [16] and have a high specificity and good sensitivity [17]. Children with higher Total Difficulties scores (TDS) in the SDQ have higher probabilities of a clinical disorder [18]. “This is true for each one point increase in TDS across the full range and is seen for the parental, teacher and youth SDQs alike” ([18], p. 100).

Other studies carried out in Israel have found that more Israeli Arab than Jewish adolescents reported their intention to seek help from teachers and educational counselors [13], and more Israeli Arab adolescents exposed to frequent and severe acts of violence requested help from a mental health professional [19].

These findings point to the importance of a better understanding of the actual skills of the school staff responsible for the Israeli Arab students, and what their potential contribution could be in meeting the needs of children and adolescents. There are relatively few school psychologists in the Arab educational system, mostly due to a dearth of Israeli Arab educational psychologists [20]. An unpublished report from the Department of Information of the Ministry of Health of Israel reveals that between the years 2000 and 2013, out of the 5664 licenses approved for clinical psychologists, only 6.2 % were granted to Israeli Arabs, while Israeli Arab children and adolescents represented nearly 26 % of all Israeli minors in 2011. Therefore, school counselors bear most of the burden of solving the students’ problems. The role of the school counselor, as determined two decades ago, included a large basket of responsibilities and purposes, such as individual counseling for personal and social adjustment, group counseling within the classroom setting, crisis intervention, improving learning skills, giving preventive education in developmental and substance abuse issues and running life skills programs [21]. However, over the years, paradigms in counseling have changed from treatment of children to focusing on prevention, and the recent shift is to a wellness paradigm [22].

Several factors have been identified as increasing the probability of help-seeking in school by adolescents in general. First, it is estimated that adolescents at high risk for emotional or behavioral problems will be more likely to seek help than those at low risk, since one of the main causes for seeking help is the adolescent’s need to deal with his/her emotional distress [8, 23]. Another factor that has been identified as encouraging help-seeking from professional mental health services is social or family support. Some studies have found that adolescents with greater levels of support at home were more willing to seek help also from their teachers and not only from friends and family members [1, 24, 25]. Well-being and satisfaction with school, family and friends have also been identified as important predictors of willingness to seek help from school sources [10]. The opposing view, however, is presented by Sears [26], who claims that “youths who sought professional help were less likely to talk to others when they have problems than those who had not sought professional help” (p. 401). Kuhl et al., [27] found that high school students who perceived that their family, friends or they themselves could deal with their problems adequately, were less likely to seek help. These findings point to the possibility that these youngsters approach mental health professionals when the family support system or their well-being at home is not strong or when family is perceived to be part of the problem and not of the solution.

The school setting, with its captive audience and access to large populations of youth, has been recognized as an important - and some would say optimal- community venue for identifying adolescents in distress, and one where primary interventions and preventive programs may be carried out to fortify children’s resilience and coping mechanisms [5, 28, 29]. This has even more relevance for minority populations that have few alternatives for mental health service use.

In spite of the advantages of identifying and caring for adolescents in distress through the school system, one main limitation remains, namely that school services cannot care for school dropouts, among whom we would probably find more pathology, and are therefore the adolescents most in need.

This paper presents findings of the Galilee Study regarding adolescents’ help- seeking practices in school. The data presented here are part of a larger epidemiological study on mental health status and structural and cultural constraints regarding help-seeking among Israeli Arab minority adolescents and their mothers, carried out in five Arab localities in the Galilee region in Israel among 9th grade school students. Although the ISMEHA study already showed a decade ago increased help-seeking in school among Israeli Arab students [8], it did so while analyzing the Israeli Arab minority as a homogenous group. The Galilee Study addresses the service needs and behaviors of minority adolescents from different socioeconomic and cultural backgrounds and thus aims to present a more nuanced picture of help-seeking in Israeli Arab adolescents.

A brief review of the characteristics of the general Arab minority in Israel reveals that they constitute 21 % of all Israeli citizens and 26.2 % among those below 18 years of age [30], and are over-represented in all indicators of poverty, distress and underdevelopment [31]. Unemployment rates are higher among Israeli Arabs than among Jews, and the school drop-out rates of Israeli Arabs are twice as high as those of Jews [32]. In 2013, 63.5 % of Arab children and adolescents lived below the poverty line, as compared with 21.6 % of Jewish minors [33].

The objectives of this paper are to examine adolescents’ help-seeking in school comparing adolescents at high risk with those at low risk for a mental disorder, in different population groups belonging to the Israeli Arab minority. Given that studies show that high risk subjects will be more likely to seek help and care [8, 23], we believe the particular characteristics and needs of these adolescents should be better understood. The questions posed were: Are adolescents at high risk for a mental disorder in these populations more likely than those at low risk to seek help in school? Whom do they consult? How does well-being at home affect help-seeking in school? Do factors such as religion and neighborhood influence help-seeking practices?

Our findings will assist us in proposing policy recommendations in the framework of the Mental Health Reform instituted in Israel in 2015.

## Methods

### The study population

The study population included all 9th grade students living in four localities in the Galilee and one in the northern Triangle. These localities are representative of the Arab localities in the north of Israel that have more than 5000 inhabitants. They include both traditional and modern localities, diverse levels of religiosity and both original residents and 1948 internal refugees [34]. The rest of the Israeli Arab citizens, not represented in this study, live in mixed Jewish-Arab cities (10 %), and in the southern Negev area (13.5 %) [4]. Israeli Arab citizens or Palestinian residents living in East Jerusalem comprise a separate socio-political population group and were not included in this study. In addition, the localities selected for this study did not have a substantial Christian population and therefore the small Christian minority will not be included in the analyses related to religious identity.

The main criterion for selecting a given locality was whether or not there was a public mental health clinic for children and adolescents. At the time this study was designed, there were two child and adolescent mental health clinics for the Arab population in the Galilee and northern Triangle regions, one in a mainly Muslim locality and the other in a mainly Druze locality. These two localities were chosen, and for comparison purposes we chose three like-sized localities, with a similar ethnic/religious composition, with no clinic. Thus we had one stratum consisting of two large cities, with a predominantly Muslim population – one with a clinic and one without–, and another consisting of three smaller towns with a largely Druze population – one with a clinic and two without. In addition to different religions (Muslim and Druze), these localities also differ with regard to socio-economic status. In the larger, predominantly Muslim localities, socio-economic status and average monthly wage for employed workers is lower than in than the smaller Druze localities ([35], table C14, pp. 100–101). In all localities, however, average wage was lower than the national average wage of NIS 8018 [36].

### The sample

#### Sampling frame

The sample was based on the register of the Ministry of Education of Israel, updated to May 2012. This register included the names of all students belonging to the cohort that was to begin the 9th grade on the 2012–2013 school year and other data such as student’s Israeli ID, birth date, name of parents and contact phone number. All 9th graders registered in school and attending class in these 5 towns were included in the study (*N* = 2366). Not included were 220 adolescents who were either: a) living in the town but had dropped out or were not registered in the school records; b) registered in the school records but did not attend school and were reported as drop-outs by the school advisor; and c) students who lived in the town but were studying out of town (private schools or other).

#### Sample size and sample probability

The target was to reach a sample of approximately 1000 Muslim and 1000 Druze 9th graders living in the selected localities, in order to produce in each stratum an estimate of the rate of mental disorders, with a 95 % confidence interval of 3 %. The sample size was calculated under the following assumptions: (1) The total rate of mental disorders in Israel, according to the ISMEHA, is 12 % [37], and (2) a rate of 12 % could be obtained if we selected for this study 39 % of the quartile of adolescents who scored highest in the screening instrument and 3 % of the remaining 3 quartiles. The design effect for cluster sampling relative to simple random sampling is 1.7.

### Measurements

*Emotional and behavioral problems* were assessed with the self-report version of the Strengths and Difficulties Questionnaire (SDQ) – Arabic version [14, 38], (http://www.sdqinfo.com).

The SDQ is a screening instrument designed for evaluating social, emotional and behavioral functioning in 4–17 year old children and adolescents [14, 15]. It includes 25 items that cover four clinical domains, namely: hyperactivity-inattention, emotional symptoms, peer-relationship problems and conduct problems, and one distinct pro-social behavior domain. Each item is rated on a 3-point scale as 0 (not true), 1 (somewhat true), or 2 (certainly true). In addition to the clinical domains, the SDQ includes an impact module that asks whether the adolescent has a problem, its degree of chronicity and whether this results in emotional distress, social impairment or burden to the family. The questionnaire has three versions (mother’s version, teacher’s version and self-version. The psychometric properties of the SDQ in Arabic have been proven satisfactory [39].

*The sociodemographic questionnaire* tapped the following data: religion of parents and adolescent, number of siblings in the family, marital status of parents, maternal education, paternal and maternal employment status and whether the family is in the care of the welfare agencies. Gender and town of residence were obtained from the Registry of the Ministry of Education, which served as the population base for the study.

*Well-being at home as a proxy indicator of family support*: The following question assessed was used as a proxy measure for family support: “To what extent do you feel comfortable at home? Adolescents could choose one of four answers: very much, somewhat, very little, not at all. Given that 84.4 % of all adolescents answered ‘very much’, the other 3 responses were collapsed and the question was dichotomized as ‘very much’ vs. ‘not much’.

*Help-seeking in school*: Four questions were posed to the adolescent:

- Did you ever feel the need to consult a mental health professional?
- Did you consult someone in school in the past year regarding issues such as problems with peers, problems at home, concentration problems or other problems not related to the school curriculum?
- Who did you actually consult in school?
- Which of the school staff seems to you the most appropriate to give advice to students regarding emotional problems or problems with family or friends? The list of sources included the school counselor, grade teacher, another teacher, psychologist, school principal, school nurse, school secretary, friends, other.

### Study design

This project was designed as a 2-stage study. The first stage, the screening stage, was carried out in the classroom and included all 9th grade students in the localities chosen. For the second stage – the follow-up stage- which was carried out in the subject’s home, a sample of those participating in the screening stage was selected, oversampling for adolescents with higher probability of having an emotional or behavioral problem according to the screening instrument used in the first stage. All adolescents in each of the 5 localities were listed in descending order according to their score in the SDQ’s Total Difficulties scale (TDS). All those in the highest 25 % of the TDS distribution in each locality were included in the sample, as well as a simple systematic sample without replacement of those in the lower 75 % with a lower risk of having a problem. This produced an oversampling of adolescents with a higher probability of having an emotional or behavioral problem in order to increase the statistical power and robustness of the analyses, as it allowed for the comparison of high-risk and low-risk adolescents with an adequate number of subjects in each category. All analyses are presented as comparing adolescents in the high risk group with those in the low risk group. The two smaller Druze localities that do not have a mental health clinic in their vicinity were analyzed as one single entity since they were very similar in their size, ethnic composition and socio-economic traits.

Both adolescents and their mothers were interviewed at home during this second stage. Mothers provided information on socio-demographic traits of the family and the adolescent provided information regarding well-being at home and help-seeking practices in school.

### Procedures

#### The screening stage

Questionnaires were completed by the adolescents in the classroom between September 2012 and May 2013. For students who were not present on the day of the data collection, second and third attempts were made to have them fill in the SDQ in the school counselor’s room as soon as they came back to school. Only students whose parents had signed an informed consent form and had turned it in were allowed to fill in the questionnaire.

#### The second stage: in-depth home interviews

The second stage was carried out using a face-to-face interview mode at the respondents’ home between October 2013 and May 2014. Adolescents and their mothers were interviewed simultaneously and independently by 2 lay interviewers in two different rooms at the home. The Rikaz Databank Center of the Galilee Society, together with the general coordinator, were responsible for data collection during the second stage, for training of interviewers, for supervising the field work and for quality control.

### Data collection

**Fig. 1:**
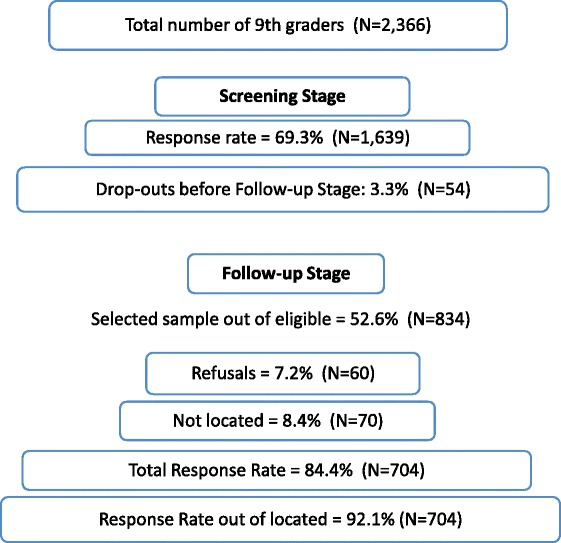
Data collection and response rates in the general study population

Figure 1 shows a flowchart of the data collection process for the general study population. It shows that response rate in the first screening stage was 69.3 % (*N* = 1639) and for the follow-up stage, among the located sample, it was 92.1 % (*N* = 704).

### Statistical analyses

Statistical analyses were conducted using an IBM SPSS-21 module (IBM-SPSS Inc. Chicago, Il). Mean SDQ scores and standard deviations are presented for the SDQ scales and the TDS. All analyses are presented comparing high risk with low risk adolescents, two groups with approximately equal number of subjects. Reports of help-seeking and wellbeing were calculated for each risk group. Pearson Chi square was applied to test the likelihood that the observed differences between the sets arose by chance. The significance level was set to equal to or below 0.05. Binary logistic regression analyses were carried out in order to predict help-seeking in school employing variables found to be significantly associated with help-seeking in the bivariate analyses. High and low risk subjects were analyzed separately.

### Findings

Table 1 shows the socio-demographic characteristics of the study population that participated in the follow-up stage (*N* = 704), and on whom our results are based. In this population there were more female than male adolescents. Half of the adolescents were Muslim, 44 % Druze and 5 % Christian. More than half had 2 or 3 siblings and one third had 4 or 5 siblings. More than 60 % of mothers had only primary school education or some high school education but with no diploma, and only 12 % had some academic studies. Seventy percent of fathers were employed. Eighteen percent of the families were under the care of the welfare services. All localities had more or less equal number of respondents and a response rate of above 90 %, except for locality 4 where the response rate was 63.5 %.

**Table 1:** Demographic characteristics of the study population

Variable	*N* (704)	% (100.0)
Gender		
Male	307	43.6
Female	397	56.4
Religion		
Muslim	358	50.9
Druze	309	43.9
Christian	37	5.3
Number of siblings		
1	27	3.8
2–3	375	53.3
4–5	242	34.3
6+	42	6.0
Missing data	18	2.6
Paternal education		
Only primary school	36	5.2
Junior high school	168	23.9
High school without diploma	181	25.7
High school diploma	127	18.0
Academic studies	103	14.7
Missing data	89	12.6
Maternal education		
Only primary school	50	7.7
Junior high school	209	29.7
High school without diploma	165	23.4
High school diploma	127	18.0
Academic studies	84	11.9
Missing data	69	9.8
Paternal employment		
Yes	491	69.7
No	148	21.0
Missing data	65	9.2
Welfare care		
Yes	129	18.3
No	558	79.3
Missing data	17	2.4
Locality		
1	189	26.8
2	191	27.1
3	195	27.7
4	129	18.3

Table 2 shows the mean scores for each of the SDQ scales by risk group. Students in the high risk group (those included in the 25 % with the highest TDS) had significantly higher mean scores on all “problem” subscales and lower mean pro-social behavior scores than those in the low risk group (those included in the remaining 75 %). However, we found differences in mean TDS scores in the high risk group by locality: mean scores were higher in the larger Muslim localities than in the smaller Druze localities (18.6 vs. 17.4, respectively) (data not on table).

**Table 2:** Students’ mean scores in SDQ Scales by risk category

SDQ Scales	Mean scores in SDQ scales					
	High risk students (25 % highest percentiles) (*N* = 357)		Low risk students (75 % lowest percentiles) (*N* = 347)			
	Mean	SD	Mean	SD	F	*p*
Total Difficulties Score	18.14	3.8	9.22	3.3	1099.1	0.000
Emotional problems	5.61	2.0	2.25	1.7	576.7	0.000
Conduct problems	3.83	1.5	1.96	1.3	319.3	0.000
Hyperactivity	5.03	1.8	2.82	1.7	290.0	0.000
Peer problems	3.68	1.7	2.20	1.4	159.1	0.000
Pro-social behavior	7.45	2.0	8.02	1.7	16.8	0.000

Table 3 demonstrates that there was a higher proportion of girls in the high risk group than in the low risk group; there were significantly more families in welfare care in the high risk than in the low risk group (*χ*2 = 7.120; *p* = 0.008); and a significantly higher proportion of adolescents in the high risk group did not feel comfortable at home, as compared with the low risk group (*χ*2 = 27.6; p=. 000). No differences were found between the high and low risk groups regarding paternal and maternal education or paternal employment.

**Table 3:** Selected characteristics of adolescents by risk category

Characteristics	Risk category		Pearson *χ*2; *p*
	High-risk group (*N* = 357)	Low-risk group (*N* = 347)	
	% (*n*)	% (*n*)	
Gender			
Male	37.0 (132)	50.4 (175)	*χ*2 = 12.959; *p* = 0.000
Female	63.0 (225)	49.6 (172)	
In welfare care			
Yes	22.7 (79)	14.7 (50)	*χ*2 = 7.120; *p* = 0.008
No	77.3 (269)	85.3 (289)	
How comfortable do you feel at home?			
Very comfortable	77.3 (272)	91.8 (312)	*χ*2 = 27.6; *p* = 0.000
Not so comfortable	22.7 (80)	8.2 (28)	

Table 4 shows that adolescents in the high risk group were more than twice as likely as those in the low-risk group to report having felt the need to seek professional help (*χ*2 = 10.43; *p* = 0.001); and almost twice as likely to have consulted a school source during the past year (*χ*2 = 14.59; *p* = 0.000) (Table 5).

**Table 4:** Help seeking for mental health concerns by risk category

Felt need to seek professional help and actual help-seeking in school	Risk Category		
	High risk group	Low risk group	Pearson *χ*2; *p*
	% (*n*)	% (*n*)	
1. Felt need to seek professional help for emotional or behavioral problems	14.0 (49)	6.5 (22)	*χ*2 = 10.43; 0.001
2. Sought help from a school source in the past year for emotional or behavioral problems	27.1 (95)	15.2 (52)	*χ*2 = 14.59; 0.000

**Table 5:** Help-seeking in school by religion, locality and well-being at home, and risk category

Variables	High risk group (*N* = 350)		Low risk group (*N* = 341)		Pearson *χ*2; *P*
	Consulted		Consulted		
	Yes (*N* = 95)	No (*N* = 255)	Yes (*N* = 52)	No (N = 289)	
	% (*n*)	% (*n*)	% (*n*)	% (*n*)	
Religion					
Muslim	30.0 (72)	70.0 (168)	16.2 (18)	83.8 (93)	*χ*2 = 7.6; 0.006
Druze	18.0 (16)	82.0 (73)	15.3 (33)	84.7 (182)	*χ*2 = 0.32; 0.608
Christian	33.3 (7)	66.7 (14)	6.7 (1)	93.3 (14)	*χ*2 = 3.6; 0.058
Locality					
1	23.2 (16)	76.8 (53)	22.2 (26)	71.8 (91)	*χ*2 = 0.02; 0.879
2	17.0 (9)	83.0 (44)	10.4 (14)	89.6 (121)	*χ*2 = 1.5; 0.213
3	34.4 (43)	65.6 (82)	13.6 (9)	86.9 (57)	*χ*2 = 9.4; 0.002
4	26.2 (27)	73.8 (76)	13.0 (3)	87.0 (20)	*χ*2 = 1.8; 0.180
How comfortable do you feel at home?					
Very comfortable	25.0 (67)	75.0 (201)	13.5 (42)	86.5 (269)	*χ*2 = 12.5; 0.000
Not so comfortable	34.5 (28)	64.6 (51)	35.7 (10)	64.3 (18)	*χ*2 = 0.00; 0.979

Among Muslim students, a significantly greater proportion in the high-risk than in the low risk group consulted a school source (30 vs. 16.2 % respectively, *p* = 0.006), while among Druze students, we found no significant difference in help-seeking between high and low-risk students. The data regarding Christian students are based on very small numbers, and do not allow for comparisons.

Only in locality 3 was there a significant difference in consultation rates between high and low risk groups: 34.4 vs. 13.6 %, respectively (*p* = 0.002). In the other localities differences in consultation rates between the two risk groups were not significant.

Among those who feel comfortable at home, rates of consulting a school source were higher in the high risk than in the low risk group (25 vs. 13.5 %, respectively, *p* = 0.000) Among students who do not feel comfortable at home, consultation rates were high, with no differences in high vs. low risk groups (34.5 and 35.7 % respectively, *p* = 0.979.

Table 6 presents binary logistic regression analyses that were carried out to predict help-seeking in school, based on independent variables found to be significantly associated with help-seeking in the bivariate analyses, namely gender, religion, welfare care and wellbeing at home. Christian students were not included in these analyses, due to small numbers. High risk and low risk adolescents were analyzed separately. For high risk adolescents, only religion (Muslim or Druze) remained significantly associated with help-seeking in school: Muslim students were 2.02 times more likely than Druze to seek help. In the low-risk group, wellbeing at home remained significantly associated with help seeking in school: students who do not feel comfortable at home were 3.3 times more likely to seek help than those who feel comfortable at home.

**Table 6:** Likelihood of help-seeking in school in high and low risk adolescents. Binary logistic regression

Variable	High risk adolescents	Low risk adolescents
	OR (95 % CI)	OR (95 % CI)
Gender		
Male (reference)	1.0	1.0
Female	1.29 (0.837–1.982)	1.21 (0.701–2.083)
Religion		
Muslim	2.02 (0.1.308–3.113)	0.95 (0.511–1.760)
Druze (reference)	1.0	1.0
Welfare		
Yes	1.19 (0.7.18–1.972)	0.91 (0.431–1.916)
No (reference)	1.0	1.0
Wellbeing at home		
Very comfortable (reference)	1.0	1.0
Not so comfortable	1.49 (0.875–2.525)	3.28 (1.563–6.862)

Table 7 presents the school sources consulted by adolescents. Among adolescents who actually consulted (*N* = 145) 43.2 % of those in the high-risk group consulted the school counselor and 37.9 % their grade teacher. Only 3.2 % consulted a school psychologist. Among the low-risk students, consultation rates were somewhat different: 42 % consulted their grade teacher, 28 % consulted the school counselor and 14 % consulted the school principal. None consulted a school psychologist.

**Table 7:** Adolescents’ preferred school sources of advice by risk group

Source of consultation among school staff	Preferred sources of consultation by risk group			
	Who did you actually consult in school? (*N* = 145)		If you were to consult, who would be the best source among school staff? (*N* = 694)	
	High-risk group	Low-risk group	High-risk group	Low-risk group
	% (*n*)	% (*n*)	% (*n*)	% (*n*)
School Counselor	43.2 (41)	28.0 (14)	32.3 (114)	26.4 (90)
Grade Teacher	37.9 (36)	42.0 (21)	34.0 (120)	38.4 (131)
Friends	–	–	19.5 (69)	17.6 (60)
Other teacher	7.4 (7)	8.0 (4)	6.5 (23)	10.3 (35)
Principal	4.2 (4)	14.0 (7)	1.7 (6)	3.2 (11)
Psychologist	3.2 (3)	0 (0)	2.8 (10)	–
Other school sources	4.2 (4)	8.0 (4)	3.4 (11)	4.1 (14)
Total	100 (95)	100 (50)	100 (353)	100 (341)

Regarding the question: “If you were to consult someone in school, who would be the best source among school staff”, both high risk and low risk adolescents ranked the grade teacher as their first choice, the school counselor as the second choice and a friend as their third choice. Ten adolescents in the high risk group (2.8 %) mentioned the school psychologist as an option.

## Discussion

This study found that being at high risk for an emotional or behavioral problem was the strongest indicator of help-seeking in school: 27 % of adolescents at high risk compared to 15 % among those at low risk sought help in school. These findings are in agreement with other studies that have found that severity of emotional or physical distress is strongly associated with help-seeking behaviors [8, 23, 40, 41]. We found that among high risk students, there were higher help-seeking rates among Muslim than among Druze (30 vs. 18 %), and this may be partly explained by the fact that Muslim students in the high risk group had higher mean TDS scores than Druze in the same risk category, possibly reflecting more distress.

Severity of emotional or behavioral disorders may be explained by lower socio-economic status [42]. In our population, higher difficulty scores were found in the larger cities (localities 3 and 4), with a mainly Muslim population, than in the medium or smaller sized Druze localities 1 and 2. As explained by Hlihel [43] a substantial proportion of the residents in these larger cities are “internal refugees”, and are dependent on salaried work as they do not have land or other resources. In 2013, the mean wages for employees in the larger Muslim cities were lower than those in the medium sized Druze towns [36]. This discrepancy in wages may reflect a real difference in economic welfare between Muslim and Druze families. In contrast to the Muslim citizens, Druze citizens are employed by the Israeli military and security establishment, where wages are relatively high. In addition, there is widespread discrimination against the Arab Muslim citizens, a non-assimilated minority [44], with a lack of development and government investment in infrastructure, education, health services and general wellbeing [44, 45]. This multiple marginalization may explain the higher rates of distress among Muslim than among Druze 9th graders.

Another very important indicator found to be associated with help-seeking in school was how comfortable the adolescent feels at home, a proxy measure for family support. Contrary to the proposition that adolescents with greater levels of support will be more willing to seek help from their teachers and not only from friends and family [1, 24], we found that the adolescents in our population responded more according to Sears [26] and Kuhl et al., [27], who found that students who perceive that their family or friends can help them deal with their problems, will be less likely to seek help. We found that more than one third of students who reported not feeling comfortable at home consulted someone in school, regardless of risk group.

An unexpected finding was that among high risk adolescents, feeling comfortable at home did not contribute to the variance in help-seeking in school over and above the risk category, while among the low risk adolescents, those who felt uncomfortable at home were 3.7 times more likely to seek help in school. This indicator of lack of support at home has emerged as an important independent indicator of help-seeking among those not classified as high risk adolescents, who might go undetected otherwise.

Among Druze students, only 18 % of those defined as at high risk for a mental disorder, consulted a school source, as compared with 30 % among Muslim students in the same risk group. These lower help-seeking practices in school among the Druze may be related in some way to their feeling more comfortable at home and to the reliance on family sources in times of distress and also to the size of the locality of residence. This may be a factor encouraging help-seeking, as in the larger cities there is relatively less familiarity between the student and the staff providing help, as compared with the intimacy between students and school staff in the smaller communities. Tishby et al., [3] addressed the complaint of students that “information in the school system tends to ‘leak’ to teachers and administrative staff, making them feel insecure about discussing personal issues with the counselors” (p.260), which is more likely to happen in smaller, closed communities.

It is possible that the students whose parents refused to participate in the study had more learning and social difficulties. In our study, teachers were requested to give an approximate estimate of the school achievement of each student and categorize them as high, medium or low achievers. We found that for 22.5 % of students who were rated as high achievers, 30.7 % of medium rated achievers and 41.2 % of low school achievers, parents refused to participate in the study. Since low school achievement has been found to be associated with more emotional and behavioral problems [46], these response rates should be considered when interpreting the results. As expected, and as a consequence of the higher response rates in locality 3, we found there more students with low achievement and with higher mean TDS than in other localities, where response rates were lower and where low achievers were less likely to participate. However, this may only partially explain the differences in mean TDS scores, since response rates in locality 4 were low but their mean TDS scores were high.

In sum, we see a constellation of factors associated with Israeli Arab adolescents who seek help in school: they are students at higher risk for an emotional or behavioral disorder, they have more socio-economic hardship, they feel less comfortable at home and they are more likely to live in the larger Muslim cities.

One remaining key question is: Who provides the needed services? We examined who the students actually consulted and who they would prefer to consult in school. It is important to add here that the educational system in Israel is segregated by ethnicity and that not all schools in the Arab sector have school counselors. Until 1999, only 20 % of Arab schools had a school counselor, as compared to 80 % of the Jewish schools [47]. Between 2000 and 2007, due to relatively intense investment in professional training, the number of school counselors in the Arab schools increased [47], although the gap still remains large and many challenges persist for the school counselor in the Arab schools, not only because of the many roles allocated to the counselor but also because of the need to work with the basic material problems of very socially disadvantaged children and adolescents [31, 47, 48].

Among high-risk students, more consulted the school counselor, followed by the grade teacher, while among the low-risk students, whose problems may be different from those of the high-risk students, more consulted the grade teacher. These findings are consistent with those of the ISMEHA study [8], which reported that the school counselors were the sources most frequently consulted by Israeli Arab adolescents: 51 % of those who needed help consulted their accessible Arabic-speaking school sources. In the Grinstein-Weiss et al. study [10], Israeli Arab students were more willing to seek help than their Jewish counterparts; the interpretation may lie in the fact that the sources of help stem from their own community and culture and therefore are both more effective and more utilized [49]. It is important to note that only 3 % in the high risk group and none in the low risk group consulted a school psychologist, the specialized mental health professional source available in the school, probably due to the fact that they are very few psychologists in the Israeli Arab educational system [20].

### Limitations

Our study sample is representative of the Muslim and Druze population living in the north of Israel but does not represent Christian Arabs, the mixed Jewish-Arab urban populations and Israeli Arabs living in the South of Israel. Further studies need to address the needs of adolescents in these populations.

A further limitation relates to the possibility of selection bias. Since there were more males and more under achieving students among non-respondents than among respondents, it is possible that our results underestimate the true school help-seeking rates. This has to be taken into consideration when planning and revising school mental health services for adolescents.

### Conclusions and policy implications

The Mental Health Reform introduced by the Ministry of Health (MOH) of Israel in 2015, transfers the responsibility for provision of mental health services from the government to the nonprofit MOH health plans [50, 51]. However, there is no government agency in charge of coordination between the Ministry of Health and the ministries involved in mental service provision to children and adolescents, namely the Ministry of Education and the Ministry of Social Affairs. As yet, no decision has been reached as to whether school mental health services should be integrated with the rest of the services provided to children and adolescents [52].

Given the important role that the school plays as a first, and sometimes sole consultation option for minority adolescents with a high risk for mental disorders, one of the possible strategies to enhance mental health service provision to these minority students, particularly in the larger and poorer Muslim localities, is to integrate the educational system within the child and adolescent mental health (CAMH) services in Israel, and to consider the option of an integrated system of mental health services in which teachers receive additional training in order to be able to recognize and deal with minor emotional and behavioral issues, and have sufficient knowledge and awareness to make referrals to the appropriate agencies for further care [52].

According to Sterne and Porter [52], this would lead to more structured service planning, including attention and more intensive services for those most in need.

In Israel the comprehensive Mental Health Reform has been implemented since July 2015, but it 50 does not articulate the linkage between the community mental health services and the school mental health services. Collaboration with the community mental health clinics for children and adolescents, as an integrated community network of care, is lacking.

As claimed by Rosen et al., [50] in the light of the new reform in Israel, not enough attention is paid to mild and moderate psychiatric problems and most of the resources of the system are directed toward a small portion of the more severely mentally ill. These mild and moderate psychiatric problems are precisely those that appear frequently among adolescents, and if they go untreated are likely to become more severe problems later on in life. Sterne and Porter [52] in their comprehensive study of mental health services for children and adolescents in Israel, emphasize the “…almost complete lack of integration, coordination and cooperation among agencies, at both the local and national levels” ([52], p.21), and the “… lack of shared language and understanding between professionals in the social and health services…” ([52], p.21).

### Recommendations

Educators and school counselors play an important role in the emotional well-being of their students, particularly those suffering from distress, who experience a lack of family support. It is therefore necessary to invest in special training for educators, to provide them with the skills necessary to identify students in need and be able to refer them to formal and professional sources for more specialized interventions. It is also important to increase the number of school counselors and school psychologists in the Israeli Arab school system, especially in middle and high schools.

An additional recommendation would be for the education authorities to create a screening system to identify students with emotional and behavioral problems. We suggest the use of the SDQ, a simple and effective tool that allows school counselors to make a preliminary identification of children with distress and emotional or behavioral difficulties [14]. As well, as found in this study, a simple question tapping wellbeing of the student at home may indicate particular needs of students who are not classified as being at high risk according to the SDQ.

The school system, however, is not equipped to deal satisfactorily with all cases of emotional disorders. Investing in mental health clinics for children and adolescents in the Israeli Arab sector is a high priority. There is a need to increase the number of these clinics and of Israeli Arab mental health professionals who share the language and culture background of these adolescents. Coordination and cooperation between the counselors and the educational staff at the school, the welfare system and the professional staff in the mental health clinic is essential.

## Abbreviations

CAMH: Child and Adolescent Mental Health
ISMEHA: Israel Survey of Mental Health among Adolescents
MOH: Ministry of Health
NIS: New Israel Shekel
PCP: Primary care practitioner
SDQ: Strengths and Difficulties Questionnaire
TDS: Total Difficulties Score in the Strengths and Difficulties Questionnaire
